# Bubble-Mediated Production of Few-Layer Graphene via Vapor–Liquid Reaction between Carbon Dioxide and Magnesium Melt

**DOI:** 10.3390/ma17040897

**Published:** 2024-02-15

**Authors:** Xuejian Li, Xiaojun Wang, Hailong Shi, Yuchao Jin, Xiaoshi Hu, Chao Xu, Lunyuan Tang, Min Ma, Liwei Lu

**Affiliations:** 1 State Key Laboratory of Advanced Welding and Joining, Harbin Institute of Technology, Harbin 150001, China; lixuejian@hit.edu.cn (X.L.); jinyuchao_1998@126.com (Y.J.); huxiaoshi@hit.edu.cn (X.H.); cxu@hit.edu.cn (C.X.); 2 Hunan Rongtuo New Material Research Co., Ltd., Xiangtan 411201, China; 3 School of Materials Science and Engineering, Hunan University of Science and Technology, Xiangtan 411201, China

**Keywords:** CO_2_ bubbles, few-layer graphene, magnesium melt, magnesiothermic reaction, growth mechanism

## Abstract

It is urgent to develop novel technologies to convert carbon dioxide to graphene. In this work, a bubble-mediated approach via a chemical reaction between carbon dioxide gas and magnesium melt to fabricate a few-layer graphene was illustrated. The morphology and defects of graphene can be regulated by manipulating the melt temperature. The preparation of graphene at 720 °C exhibited an excellent quality of surface and graphitization degree. The high-quality few-layer graphene can be grown under the combined effect of carbon dioxide bubbles and in-situ grown MgO. This preparation method possesses the advantages of high efficiency, low cost, and environmental protection, which may provide a new strategy for the recovery and reuse of greenhouse gases.

## 1. Introduction

With the proposal of the double-carbon policy, it is urgent to develop efficient utilization technologies for carbon dioxide (CO_2_) [1,2,3]. Carbonaceous nanomaterials (NMs) possess enormous potential in applying electrochemical energy storage and nanocomposites, and converting CO_2_ into usable carbonaceous NMs has become a feasible method for the recovery of greenhouse gases [4,5,6]. However, it is very difficult to break the stable C-O bond of CO_2_ due to the high bond energy of 799 kJ/mol [7]. At present, the resource utilization of CO_2_ gas can be achieved via chemical reactions with alkali metals (such as Li [8], Na [9], and K [10]). Unfortunately, these highly chemically active alkali metals need to be stored in a dry environment, resulting in many additional challenges in safety and cost. In contrast, magnesiothermic reactions using metal magnesium (Mg) as a reducing agent are ancient and safe chemical reduction methods, which are particularly effective in breaking down strong chemical bonds such as C=O in CO_2_ and S=O in SiO_2_ [11]. Therefore, using chemical reactions between Mg and CO_2_ gas to prepare carbonaceous NMs may be a meaningful choice.

In 2011, Chakrabarti et al. [12] explored the conversion of CO_2_ into few-layer graphene by igniting the Mg ribbon in dry ice. This work provided an innovative idea for producing one of the most promising carbonaceous NMs by capturing CO_2_ gas. After this work, Zhang et al. [13] reported a method for controlling the shape and dimensions effectively by manipulating the reaction temperature, which achieved controlled synthesis of carbonaceous NMs including carbon nanotubes, mesoporous graphene, and hollow carbon nanoboxes. Most recently, Ji et al. [14] developed a modified magnesiothermic reaction to prepare porous graphene with a high specific surface area and great conductivity by using a Zn and Mg mixture as a reductant. Although the above methods based on magnesiothermic reactions can achieve the preparation of carbonaceous NMs, it is difficult to achieve large-scale and controllable preparation simultaneously due to the raw material being Mg powder. Thus, it is necessary to optimize the magnesiothermic reactions to achieve the batch-controllable preparation of carbonaceous NMs for CO_2_ storage and utilization.

A typical synthetic strategy (CVD) for few-layer graphene mostly relies on a “hard” template such as metal copper (Cu) or metal nickel [15,16]. However, the method still suffers from high consumption of substrates and quality degradation of few-layer graphene due to the removal of the “hard” template via an etching process. Thus, the biggest problem of using CVD to produce graphene is the complex process (low efficiency) and the consequent high cost. Recently, many studies have shown that bubbles as a new tool have a significant impact on the production of few-layer graphene (bubble-mediated technique); the bubbles can create a large amount of gas/liquid or gas/solid interfaces within a limited space [17,18,19]. Because of their unique morphological characteristics, bubbles can be used as the favorite interface for graphene growth and as a driving force for breaking graphite layers to prepare graphene at high temperatures. Thus, bubble-mediated technology represents a new idea with the potential to achieve the controllable and efficient preparation of graphene. For example, Tang reported a chemical vapor deposition method using Cu melt as the catalyst for the mass production of high-quality graphite. Bubbles containing natural gas or CH_4_ are produced by introducing an aerator into Cu melt. The graphite was grown on bubble surfaces with a thickness ranging from a few to 40 graphitic layers [20]. In addition, a CO_2_ bubble-mediated production of graphene via a vapor–liquid reaction in Mg melt was realized in our previous research to fabricate graphene. The input of CO_2_ bubbles can make the reaction environment more stable so that the quality of graphene can be controlled effectively [10,11,12,13,14,15,16,17,18,19,20,21,22,23]. However, the regulation and growth process of graphene should be further researched and explored in detail.

In this work, a bubble-mediated approach via a chemical reaction between CO_2_ and Mg melt to produce few-layer graphene was further studied. The morphology and defects of few-layer graphene can be regulated by manipulating the melt temperature. The as-fabricated few-layer graphene shows outstanding surface quality and graphitization degree. This article aims to explore an efficient technology for the recovery of greenhouse gases and promote the application of few-layer graphene.

## 2. Experimental Details

### 2.1. Methods

**Figure 1:**
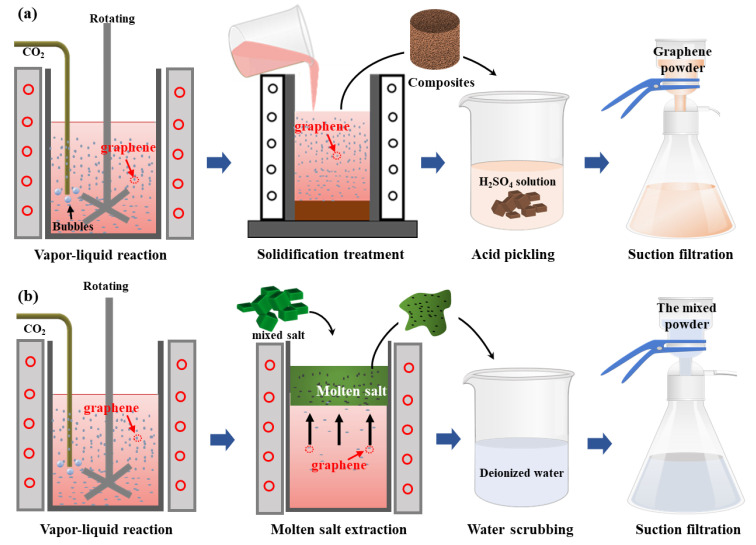
The schematic of the bubble-mediated production of few-layer graphene: (**a**) Few-layer graphene powder; (**b**) The mixture of few-layer graphene and MgO.

In a typical experiment, the schematic of the bubble-mediated production of few-layer graphene in Mg melt is shown in Figure 1a. Firstly, a pure Mg sample (600 g) was added into the stainless-steel crucible at 720 °C under the protection of CO_2_ and SF6. After the pure Mg sample was melted fully, the melt temperature was adjusted to 680–740 °C. Then, high-purity CO_2_ gas at 300 CCM was introduced into the pure Mg melt. Here, Mg melts as a reducing agent reacts with CO_2_. As the reaction proceeds, the production of few-layer graphene and MgO leads to an increase in the viscosity of the melt. CO_2_ gas cannot react effectively with Mg melt, resulting in a decrease in the yield of few-layer graphene. Thus, the chemical reaction was carried out for 20 min to ensure the uniformity of the product. Subsequently, the few-layer graphene/Mg composites were obtained after solidification treatment. Finally, we etched few-layer graphene/Mg composites by diluting the H_2_SO_4_ solution to remove MgO and residual Mg matrix. Few-layer graphene powder (5.8 g) can be obtained after repeated washing and drying. In contrast, to study the relationship between in situ MgO and few-layer graphene, a mixed salt of 50 wt.% NaCl and 50 wt.% KCl is added to Mg melt to extract reaction products, as shown in Figure 1b. As the mixed salt melts and is evenly dispersed in the Mg melt, few-layer graphene and MgO will float above the Mg melt with the molten salt. Then, a suspension containing MgO and few-layer graphene was obtained by dissolving the mixed salt in deionized water. Finally, the mixture including few-layer graphene and MgO was obtained after repeated washing and drying.

This reaction is carried out step by step with the continuous introduction of bubbles, instead of releasing a large amount of heat in an instant. Therefore, a stable-temperature environment can be maintained, which promotes the uniform quality of graphene in the reaction process. However, the generation of few-layer graphene in Mg leads to a significant increase in viscosity, resulting in CO_2_ gas cannot effectively feed into the melt. As a result, the yield of graphene is limited.

### 2.2. Materials Characterizations

To analyze the structural characteristics of few-layer graphene, X-ray diffraction was carried out on an X-ray diffractometer with Cu Ka radiation (XRD, Paralytical-Empyrean). The surface morphology of the as-fabricated few-layer graphene was observed using a scanning electron microscope (SEM, Zeiss-SUPRA55) and a transmission electron microscope (TEM, JEM-2100). The defect density was studied using the Raman spectrum (B&WTEK-BWS435–532SY) with the 532 nm laser wavelength. The defect degree (I_D_/I_G_) can be obtained via the intensity ratio of D peak to G peak. The G peak of few-layer graphene was normalized to ensure that Raman results could be compared with each other. An atomic force microscope (AFM) was conducted on a Bruker Dimension Fastscan to analyze the thickness of few-layer graphene. X-ray Photoelectron Spectrometer (XPS, ESCALAB-250Xi) was used to analyze the bonding characteristics of the few-layer graphene.

## 3. Results and Discuss

### 3.1. Characterizations of As-Fabricated Carbon Product

**Figure 2:**
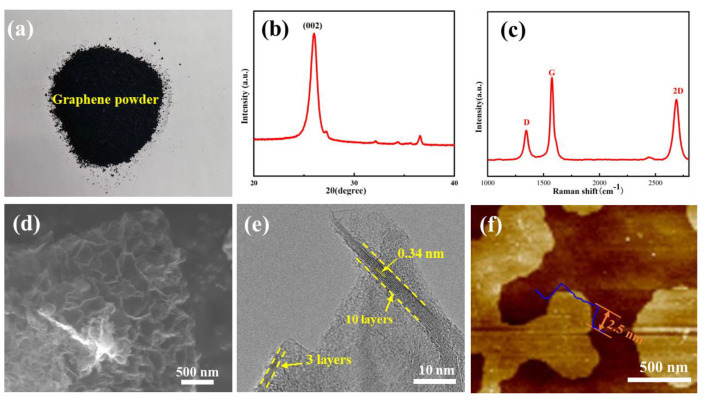
The characterizations of few-layer graphene powder: (**a**) Optical image; (**b**) XRD pattern (**c**) Raman spectrum; (**d**) SEM image; (**e**) HR-TEM image (The dotted line represents the number of graphene layers); (**f**) AFM image (The blue line represents corresponding height measurements).

Figure 2a shows the optical image of the obtained carbon product after etching off the Mg alloy when the chemical reaction was carried out at 720 °C. The morphology and structure of the as-fabricated carbon product were verified via varied means of characterization tools. The XRD result of the as-fabricated carbon product is presented in Figure 2b. The (002) characteristic peak at 26° proves that the carbon product possesses a typical graphitic feature with a high crystalline degree. Furthermore, Figure 2c shows three typical characteristic peaks at 1352 cm^−1^, 1581 cm^−1^, and 2683 cm^−1^, corresponding to the D peak, G peak, and 2D peak of few-layer graphene, respectively. It is widely known that the G peak is usually caused by the in-plane vibration of sp^2^ hybrid carbon atoms, reflecting the crystallinity and symmetry of as-fabricated few-layer graphene. The D peak is caused by the bond length, bond angle, and vacancies within the surface of few-layer graphene. The 2D peak is very sensitive to the c-axis superposition of the few-layer graphene layer. The appearance of the 2D peak in Raman spectroscopy shows the transition of the carbon product from amorphous carbon to the graphitized phase. Although high-crystallinity graphite also produces 2D peaks, the position of the 2D peak in few-layer graphene shows a clear trend of moving towards a lower wave number (blue shift phenomenon) [24,25,26,27]. These results confirm that high-crystallinity few-layer graphene can be successfully synthesized during the vapor–liquid reaction process. The SEM evidenced that the surfaces of carbon products have obvious 2D morphology with plenty of ripples and wrinkles, as shown in Figure 2d. The two-dimensional morphology of carbon products can also be determined through high-resolution TEM, as shown in Figure 2e. It can be determined that the obtained carbon product possesses about 6–10 layers. To further analyze the morphology and thickness of the as-prepared few-layer graphene, as shown in Figure 2f, the AFM image of depositing few-layer graphene dispersed in ethanol onto a silicon substrate also shows that the few-layer graphene possesses obvious two-dimensional morphology characteristics. The thickness measurement on its two-dimensional surface reveals that the thickness of the few-layer graphene fluctuates around 3 nm, indicating that the vapor–liquid reaction method is not a single-layer few-layer graphene but a few-layer graphene nanosheet with a layer number of about 10 layers. Meanwhile, the fluctuation in its thickness reflects that the surface of as-fabricated few-layer graphene contains some wrinkles. Thus, it could be fully proved that the in situ carbon product is a multi-layer few-layer graphene based on the above morphology and structural characteristics.

### 3.2. Defect Density of As-Fabricated Few-Layer Graphene

**Figure 3:**
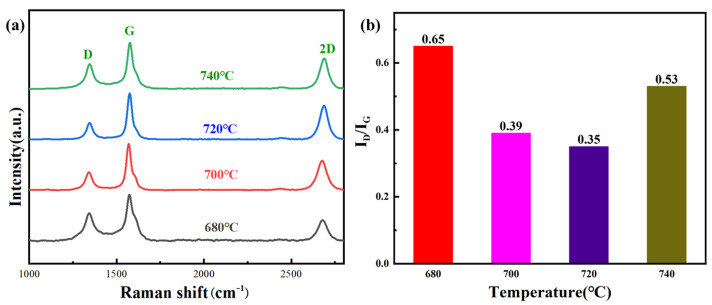
The defect of few-layer graphene: (**a**) Raman spectrum (Black, red, blue, and green line represent the temperature of graphene growth at 680 °C, 700 °C, 720 °C and 740 °C); (**b**) *I_D_*/*I_G_* value.

Figure 3a shows the Raman spectroscopy of few-layer graphene synthesized in the Mg melt temperature range of 680–740 °C. The defects of few-layer graphene can be quantitatively studied using the ratio of D peak to G peak (*I_D_*/*I_G_*) in Raman spectroscopy. The higher value of *I_D_*/*I_G_* indicates that the surface of few-layer graphene contains more defects. It can be observed that there are significant differences in the *I_D_*/*I_G_* values of few-layer graphene at various Mg melt temperatures as shown in Figure 3b, indicating that the temperature has a significant impact on the decomposition of CO_2_ gas and the crystallization process of carbon atoms. When few-layer graphene is synthesized at 680 °C, the *I_D_*/*I_G_* value calculated via Raman spectroscopy is approximately 0.65. As the temperature gradually increases, the defect density on the surface of few-layer graphene continuously decreases. When the temperature increases to 720 °C, the degree of surface defects decreases to the lowest (0.35). The above results indicate that as the temperature of the Mg melt increases, the crystallinity of the few-layer graphene generated in situ gradually improves. Under these conditions, the as-fabricated few-layer graphene possesses a larger crystalline domains and a lower density of defects [28,29]. However, as the melt temperature further increases to 740 °C, the number of defects on the surface of few-layer graphene further increases. The reasons for the changes in defect density of few-layer graphene at different temperatures will be discussed in detail in the following text.

**Figure 4:**
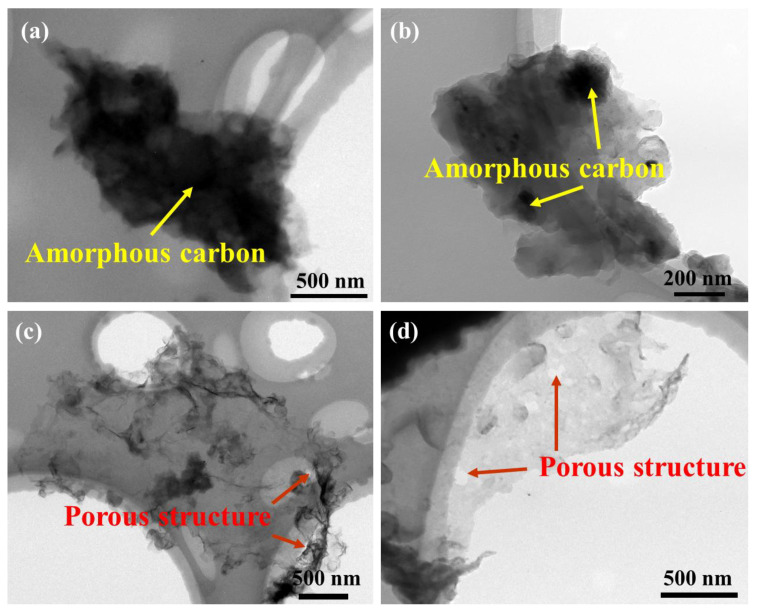
TEM images of few-layer graphene powder at different temperatures: (**a**) 680 °C; (**b**) 700 °C; (**c**) 720 °C; (**d**) 740 °C.

Figure 4 shows TEM images of obtained few-layer graphene powder synthesized at different melt temperatures. The carbon products exhibit typical two-dimensional morphology characteristics. In addition, numerous wrinkles were observed on the surface of few-layer graphene at different temperatures. When the temperature of the Mg melt is 680 °C the surface of few-layer graphene is relatively rough and covered with a layer of amorphous carbon with poor transparency. As the temperature of the Mg melt increases the content of amorphous carbon decreases significantly. When the temperature increases to 720 °C the amorphous carbon on the surface of the few-layer graphene disappears. In addition, a porous structure can also be observed on the surface of few-layer graphene at 720 °C. When the temperature of the Mg melt further increases to 740 °C the size and number of pores on the surface of few-layer graphene increase significantly. Thus, it can be determined that few-layer graphene has good crystallinity and exhibits higher surface quality when the temperature of the Mg melt is 720 °C. The changes in defects of few-layer graphene may be related to the morphology characteristics.

In the vapor–liquid reaction system, low melt temperatures will possess a low degree of crystallization, so the surface of few-layer graphene is covered with a layer of amorphous carbon. As the temperature of the magnesiothermic reactions increases the crystallinity increases and the amorphous carbon decreases on the surface, which can lead to the surface morphology becoming smoother gradually. In addition, as the temperature of Mg melt increases, CO_2_ gas may undergo chemical reactions with the surface of few-layer graphene because Mg is extremely reactive, which can accelerate the disappearance of amorphous carbon on the surface of graphene. Xing et al. found that CO_2_ gas has a dual role in the reaction process. CO_2_ gas can not only act as a reactant but can also etch the surface of graphene [14]. Zhang et al. successfully achieved the preparation of ultra-clean graphene by utilizing the selective etching effect of CO_2_ gas on amorphous carbon [30]. This effect can be described using the following equation:

CO_2_ (g) + C (s)→2 CO (g).

**Figure 5:**
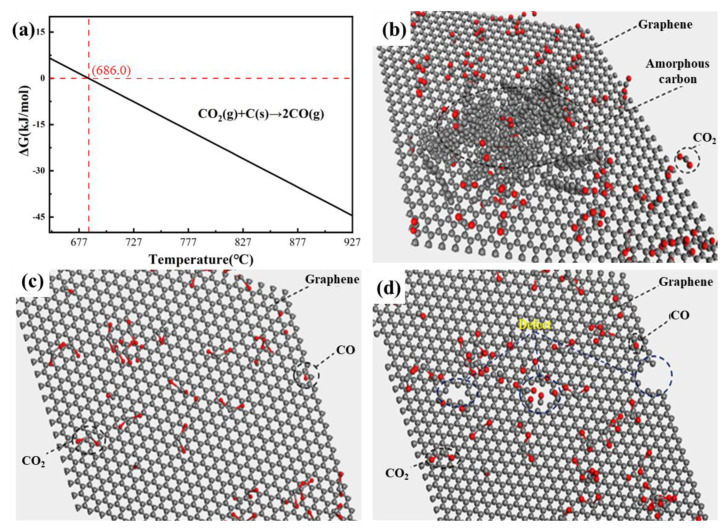
(**a**) Calculation of molar Gibbs free energy for the reaction between C and CO_2_ (The red line represents the reaction temperature at which the Gibbs free energy is zero). (**b**–**d**) Schematic diagram of CO_2_ etching process.

The specific reaction temperature between C atoms and CO_2_ gas needs to be determined through thermodynamic calculations, as shown in Figure 5. It can be seen that the molar Gibbs free energy is less than 0 when the temperature is greater than 686 °C. Thus, there is still some amorphous carbon on the surface of few-layer graphene due to poor crystallinity at 680 °C in Figure 5b. As the melt temperature gradually increases, the amorphous carbon on the surface of few-layer graphene will be first etched by CO_2_. Thus, the degree of defects in few-layer graphene gradually decreases due to the increase in the crystallinity of few-layer graphene and the CO_2_ etching effect in Figure 5c. However, as the temperature increases from 720 °C to 740 °C, the surface of few-layer graphene is no longer covered by amorphous carbon and the localized etching effect becomes more severe, causing the surface of crystallized few-layer graphene to be etched and generate some microporous structures in Figure 5d. At this synthesis temperature, more defects will be observed during Raman spectroscopy. From the above results, the melt temperature of the vapor–liquid reaction is 720 °C; the generated few-layer graphene has a lower defect density and higher surface quality.

### 3.3. Analysis of the Growth Mechanism of Few-Layer Graphene

**Figure 6:**
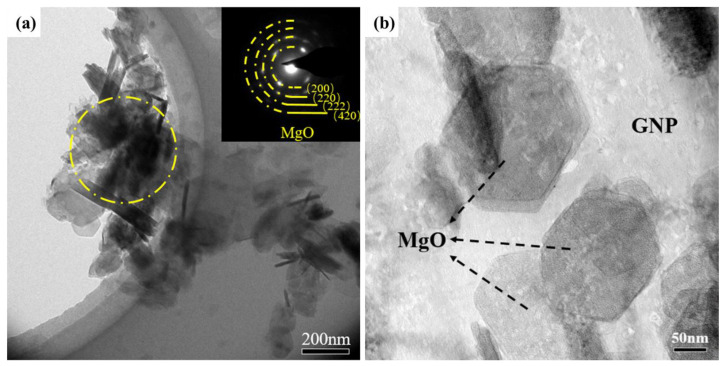
TEM bright field image of MgO and few-layer graphene (The dark contrast inside the yellow circle is MgO). (**a**) Low magnification, (**b**) High magnification.

In the vapor–liquid reaction system, the CO_2_ bubbles can be formed through the pipeline in the Mg melt. Thus, the decomposition of CO_2_ gas and the growth of few-layer graphene occur at the interfacial area between Mg melt and CO_2_ bubbles. The bubbles can serve as soft templates for the growth of few-layer graphene at high temperatures, so the reaction products can exhibit a two-dimensional morphology. In addition, it is worth noting that the surface of few-layer graphene is not a perfect two-dimensional structure but contains many wrinkles. Through the process of the Mg-CO_2_ chemical reaction, it can be found that MgO and few-layer graphene are always produced simultaneously. Accordingly, MgO in Mg melt may also have an impact on the growth of few-layer graphene. In previous studies, Zhu et al. [31] demonstrated that few-layer graphene can grow on the surface of MgO crystals controllably, which proves the hard template effect of MgO. In this experiment, the position relationship between few-layer graphene and MgO was studied to verify the effect of MgO. The mixed powder including MgO and few-layer graphene was extracted using molten salt. As shown in Figure 6a, with the growth of few-layer graphene on the surface of bubbles, the existence of MgO nanoparticles on the surface of few-layer graphene can also be confirmed via a selected area electron diffraction pattern. Furthermore, MgO particles distributed on the surface of few-layer graphene can also be directly observed at high magnification in Figure 6b.

**Figure 7:**
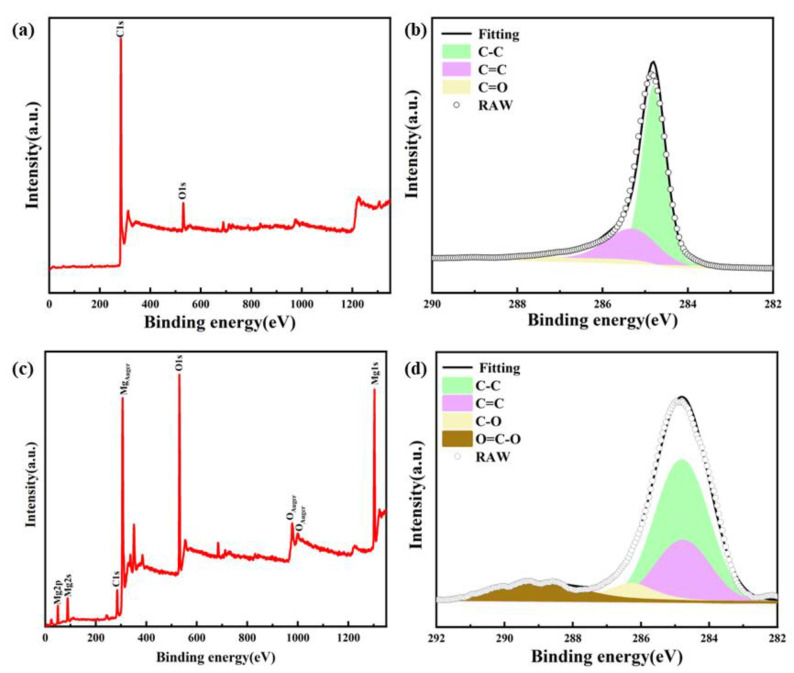
(**a**) XPS survey spectrum of pickling few-layer graphene powders; (**b**) C1s spectrum of pickling few-layer graphene powder; (**c**) XPS survey spectrum of the mixture of MgO and few-layer graphene powders; (**d**) C1s spectrum of the mixture of MgO and few-layer graphene powders.

To further analyze the bonding characteristics of few-layer graphene and MgO, XPS testing was conducted on few-layer graphene powders, as shown in Figure 7. Figure 7a shows the two elements, including Carbon (C) and Oxygen (O), that can be observed. Furthermore, the C1s spectra of few-layer graphene powder can be divided into three patterns at 284.8 eV, 285.3 eV, and 286.4 eV, corresponding to sp^2^ hybridized carbon, sp3 hybridized carbon, and the C-O functional group. In comparison, the result shows that the reaction products obtained via the molten salt extraction method have +2 valence Mg atoms and higher peak O1s, which represents the MgO products obtained using the molten salt extraction method as shown in Figure 7c. In addition, except for the sp^2^ hybridized carbon, sp^3^ hybridized carbon, and C-O functional group, carboxyl functional groups(-C=O-) can be found to exist in the mixture (288.9 eV) as shown in Figure 7d. The -C=O- bond requires positive ions to participate, while only +2 valence Mg can play this role in the reaction system. Thus, few-layer graphene has a certain chemical binding with the coexisting MgO during the growth process. The above results can prove that few-layer graphene and MgO are interdependent in growth, and in situ MgO can serve as another template for few-layer graphene growth.

**Figure 8:**
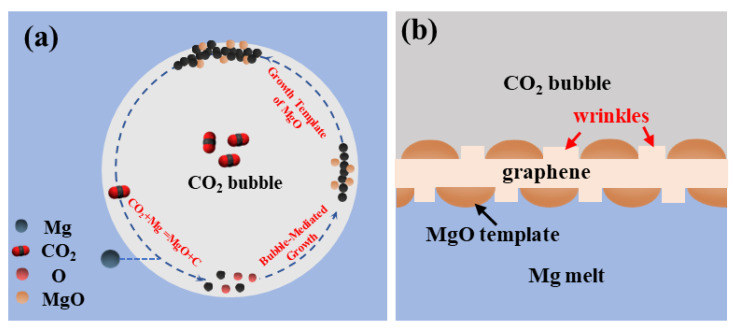
The growth process of in situ few-layer graphene. (**a**) The schematic diagram of few-layer graphene growth in the2 gas–liquid reaction, (**b**) The formation process of wrinkles on the surface of graphene.

Based on the above results, the growth step of as-fabricated few-layer graphene in the vapor–liquid reaction of Mg-CO_2_ can be inferred as follows. First, Mg melt reacts with CO_2_ molecules to generate carbon atoms and MgO. The carbon atoms connect and form bonds to form amorphous carbon and few-layer graphene products. Additionally, Mg- CO_2_ releases a large amount of heat during the reaction, which can cause the amorphous carbon to undergo structural transformation to form few-layer graphene microcrystals at high temperatures [20,28]. The growth of few-layer graphene is achieved via the interconnection of few-layer graphene microcrystals or the epitaxial growth of carbon atoms connected via the edges of few-layer graphene microcrystals. The schematic diagram of few-layer graphene growth in the Mg–CO_2_ gas–liquid reaction is shown in Figure 8a. With the growth of few-layer graphene on the vapor–liquid interface, MgO nanoparticles can be coated on the surface of few-layer graphene simultaneously. The MgO on the surface of few-layer graphene can also affect the morphology of few-layer graphene [32,33,34]. The reaction products of MgO nanoparticles can serve as another template for the subsequent growth process of few-layer graphene. Thus, the surface of few-layer graphene will form the obvious wrinkles shown in Figure 8b.

## 4. Conclusions

In conclusion, a CO_2_ bubble-mediated approach in Mg melt for fabricating a few-layer graphene was illustrated. The influence of temperature on the quality and morphology of as-fabricated few-layer graphene was analyzed. The 2D morphology and growth mechanism of as-fabricated few-layer graphene were clarified. The main conclusions are as follows:

(1) A bubble-mediated approach was adopted by a chemical reaction between CO_2_ and Mg melt to fabricate a few-layer graphene. This preparation method possesses the advantages of high efficiency, low cost, and environmental protection compared to the CVD method, which will provide a new strategy for the recovery and reuse of greenhouse gases.

(2) It can be determined that few-layer graphene has good crystallinity and exhibits higher surface quality when the temperature of the Mg melt is 720 °C, which is related to the increase in crystallinity of few-layer graphene and the CO_2_ etching effect.

(3) In the vapor–liquid reaction process, the bubbles can be employed as the template for few-layer graphene growth at a high temperature, so the reaction products can exhibit a two-dimensional morphology. In addition, the in situ MgO nanoparticles can serve as other templates for the generation of subsequent reactions so that three-dimensional few-layer graphene with folded and wrinkled structures can be generated.

## Data Availability

Data are contained within the article.
